# Intrasubstance Tear of the Short Head of Biceps With Musculocutaneous and Median Nerve Compression

**DOI:** 10.5435/JAAOSGlobal-D-19-00074

**Published:** 2019-12-11

**Authors:** Harold I. Salmons, William J. Warrender, Shelby Smith, Kenneth A. Kearns, Adam Strohl

**Affiliations:** From the Department of Orthopaedics at Thomas Jefferson University, Philadelphia, PA (Mr. Salmons, Dr. Warrender, and Ms. Smith), and Philadelphia Hand to Shoulder Center, King of Prussia, PA (Dr. Kearns and Dr. Strohl).

## Abstract

Traumatic intrasubstance ruptures of the biceps brachii are rare. Injury to its tendinous insertion or origin is most common. Isolated short head injuries are rare, and musculocutaneous nerve involvement has been reported for these injuries. We present a unique case report of a young, healthy man who sustained a symptomatic median and musculocutaneous nerve compression resulting from an intrasubstance tear of the short head of the biceps after a snow blower injury. Short belly rupture with injury-associated scar tissue compression of both the median and musculocutaneous nerves was identified in the operating room. Microsurgical decompression and tendon repair with a modified Mason-Allen configuration using 0-Vicryl suture were done. By 11 months postoperative, the patient experienced resolution of his arm pain, extension and flexion improvement from 0 to +140 to +15 to +150, elimination of the Tinel sign and of concomitant arm deformity, and improvement of elbow strength.

Traumatic intrasubstance ruptures of the biceps brachii are rare and historically ascribed to military static line parachuting.^1^ The biceps brachii is commonly injured at its tendinous insertion and origin,^2^ but musculotendinous injuries have also been described.^3^ The intra-articular tendon of the long head of the biceps brachii is the most commonly injured structure followed by its extra-articular portion, at the myotendinous junction, and then within the intramuscular substance.^2,4^ The mechanism common to these injuries involves active forward flexion of the shoulder while the arm is extended.^3^ By contrast, very few cases of isolated short head injury have been identified. In a previous review, two cases of isolated short head pathology were identified, one partial and one complete.^4^ Previous reports documented traumatic rupture of the short head in two healthy young adults after a car accident and while water skiing.^5,6^ In the literature, musculocutaneous nerve impingement was reported for such injuries.^7^ To the best of our knowledge, there has never been a documented symptomatic median and musculocutaneous nerve compression after an intrasubstance tear of the short head of the biceps. We present the unique case of a complete, isolated, and traumatic rupture of the biceps short head muscle belly with musculocutaneous and median nerve compression after a snow blower injury with improvement in function and strength after surgical repair.

## Case Report

### History of Present Illness

A 26-year-old healthy man was evaluated for a snow blower-related injury in which he sustained direct trauma while under traction to his right upper extremity resulting in a mangled hand. Surgery to reconstruct the salvageable areas of the hand was done during his index admission. At his third follow-up visit from hand surgery, 6 weeks after the accident, the patient reported persistent, focal sharp pain about the elbow, weakness on elbow flexion and overhead activities, and ecchymosis in the medial aspect of his upper arm with no numbness or paresthesias. He denied any pre-existing injuries in the area.

### Physical Examination

**Figure 1:**
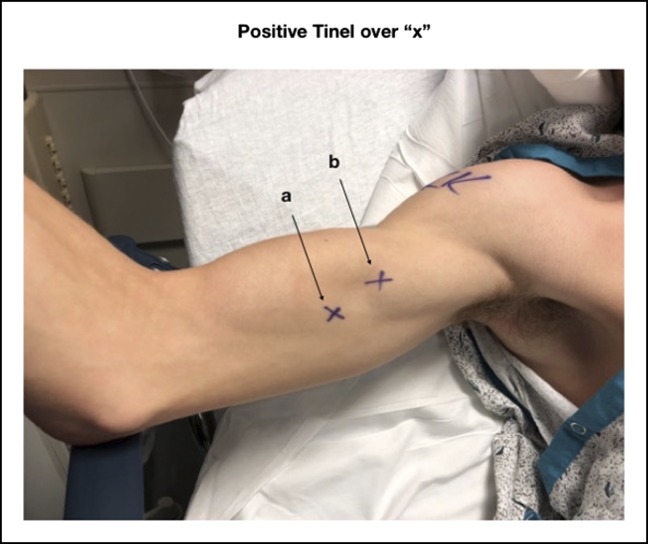
Preoperative image of patient's right arm in flexion and external rotation. Patient had a soft-tissue defect with a Tinel sign over the median nerve (a) and the musculocutaneous nerve (b) as marked by “X.”

Examination identified a soft-tissue, tender, defect in the medial aspect of the right biceps, more obvious with contraction. Shoulders were symmetric in strength and range of motion with no provocative signs. He endorsed pain with resisted elbow flexion but had negative hook and drop-arm testing. Range of motion of the elbow was zero degrees of extension to 140 of flexion with 90° of pronation and supination versus +10/150 for the unaffected left elbow. There was 4/5 strength with resisted flexion likely due to pain rather than true weakness, and the distal biceps tendon appeared intact. He was neurovascularly intact distally but had a Tinel sign over the area of tenderness (Figure 1). Electromyography was not obtained because his symptoms were clinically believed to be emanating from the severe injury in his hand rather than being better explained by a proximal nerve injury.

### Imaging

**Figure 2:**
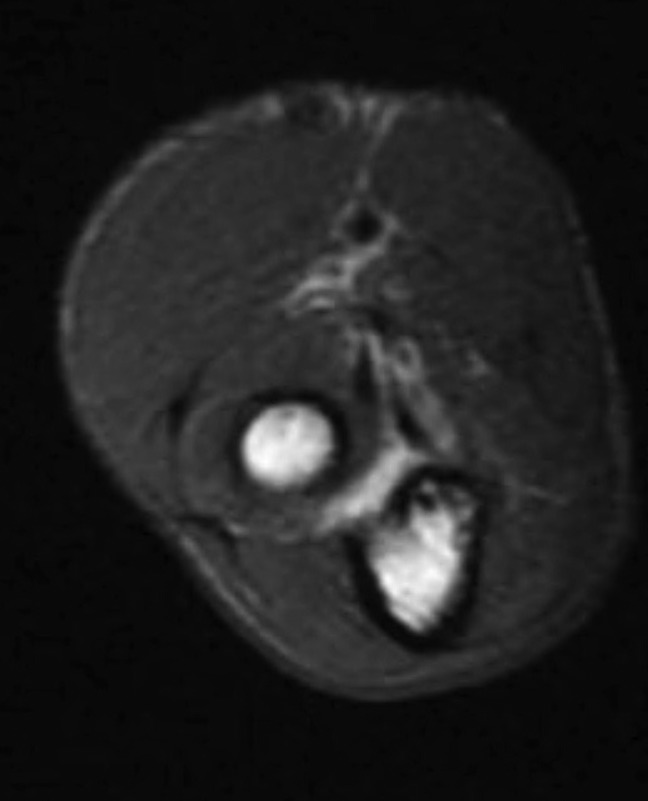
Axial T2 1.5-Tesla MRI of the right upper arm showing an intact distal biceps tendon. The image was found to be out of plane with some motion artifact, thus obscuring the injury to the short head of the biceps discovered intraoperatively.

1.0-Tesla MRIs of the shoulder and humerus were done at another institution and disclosed no tendinous, muscular, or nervous injury about the upper shoulder, but did suggest a retracted tear of the distal biceps tendon with surrounding edema at the junction of the middle to distal third of the humerus. Clinically, an intact distal biceps tendon was palpable in the antecubital fossa of the affected arm. In addition, the patient's upper-medial arm pain, ecchymosis, and Tinel sign suggested injury elsewhere. To address the discordance between the patient's initial MRI findings with our clinical examination, repeat MRI was ordered at our institution at a higher 1.5-Tesla resolution. Dynamic ultrasonography was considered but not ordered due to scheduling delays and results equivocal to MRI. Our institution's humeral and shoulder MRI series were read as normal by two board-certified musculoskeletal (MSK) radiologists, with preserved muscle bulk and signal, intact tendons without tendinosis or tear, no edema, or neurovascular structure damage identified (Figure 2).

MRI of the shoulder showed no fractures, dislocations, or acute osseous abnormalities.

### Surgical Indication

The decision to operate on the upper arm of this patient was made on the grounds of physical examination findings because the patient's symptoms did not parallel the negative reads by our institution's radiologists. At the time of the initial injury, the patient's mangled hand drew primary focus as it required emergent surgery. Given the result of the hand injury, the dull pain and symptoms initially experienced in the upper arm were attributed to pain radiating from his severe hand trauma. After hand repair, months of conservative treatments targeting his shoulder discomfort and hand recovery were pursued and failed. During that time, the patient's swelling reduced which better highlighted the medial defect of his bicep injury. Six months after initial injury, the patient's failure of conservative management, persistent inability to do activities of daily living, upper-medial arm deformity, negative Hook and drop arm testing, positive Tinel sign over the deformity, and general symptomatology led to a decision to schedule surgery for what was believed to be a right corachobrachialis rupture. In this time, a good rapport was established with the patient, who indicated agreement to proceed with this second surgery having had sufficient time to recover from his hand surgery.

### Surgical Procedure and Intraoperative Findings

**Figure 3:**
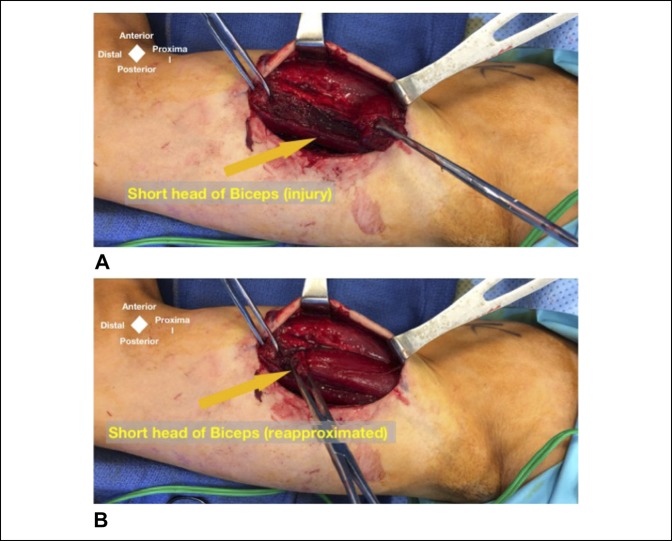
Intraoperative exposure of the injury revealed an injured short head of the biceps (**A**) and is shown reapproximated in (**B**).

**Figure 4:**
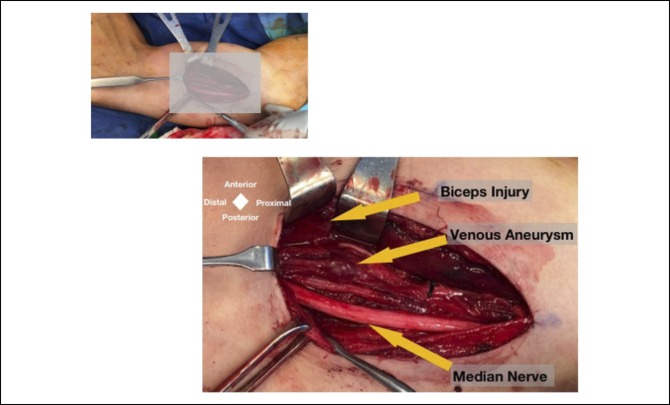
Intraoperative image showing the biceps injury, venous aneurysm, and narrowed median nerve noted to be in line with the venous injury.

**Figure 5:**
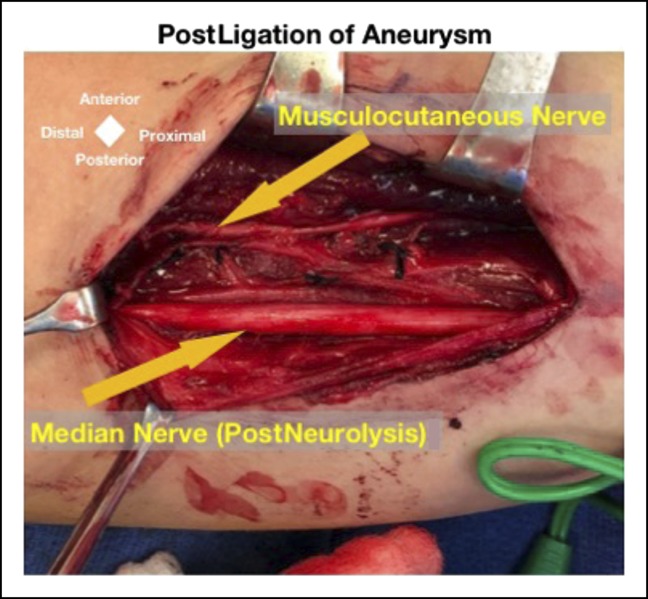
After ligation of the venous aneurysm and neurolysis, the median nerve is shown to be less narrowed, with the musculocutaneous nerve also in view.

A longitudinal incision was made in the medial-brachial bicipital groove. The basilic vein and the AP divisions of the medial antebrachial cutaneous nerve were identified. In the anterior field, the injured muscle was in fact the belly of the short head of the biceps and not a retracted tear of the distal biceps tendon as the community center's MRI suggested (Figure 3). Most of the short head of the bicep was intact to its tendinous portion distally. Further dissection identified the intact coracobrachialis muscle as it inserted onto the midhumerus with the musculocutaneous nerve found piercing through the muscle and its branches to the brachialis and the biceps. Distally, this nerve was entrapped in the scar tissue around the rupture site of the partial short head tear. The median nerve had a transverse band arranged perpendicular to the direction of its fibers. A grape-like structure was found compressing the musculocutaneous nerve in line with the transverse band that was located over the median nerve along with the ruptured portion of the biceps (Figures 4 and 5). It was likely that there was external, direct trauma to this area during his initial injury. The presumed venous aneurysm was then ligated. Microsurgical neurolysis of the median and musculocutaneous nerves was done using the operating microscope.

**Figure 6:**
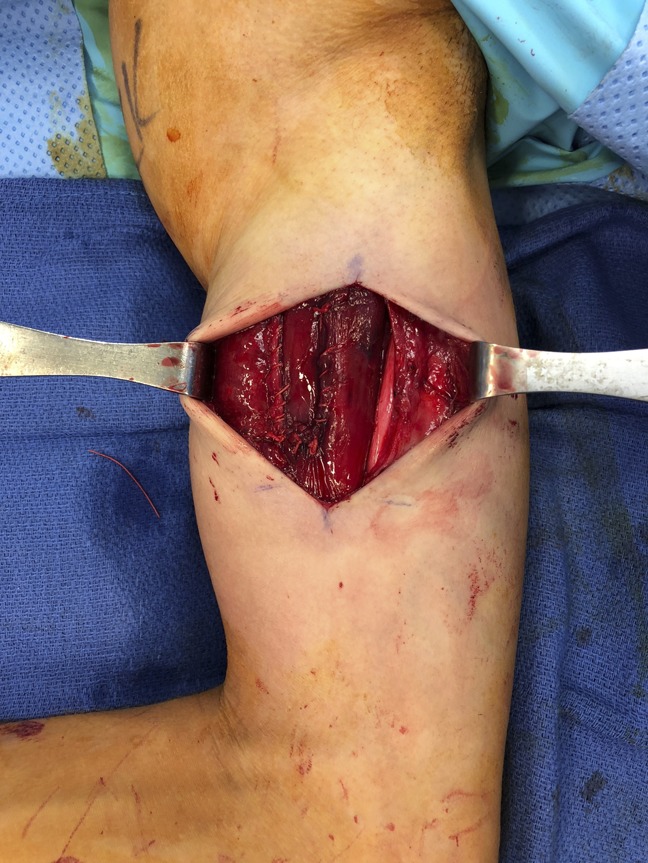
Intraoperative image showing an intact, short head of the biceps muscle after repair with three 0-Vicryl modified Mason-Allen suture configurations with a running 0-Vicryl suture oversew.

To begin repair of the short head of the biceps, the muscle was retracted proximally and scar tissue was removed. The proximal and distal ends of the tear were reapproximated and repaired through three 0-Vicryl modified Mason-Allen suture configurations. We then oversewed the repair to the intact biceps with a running 0-Vicryl suture both medially and laterally (Figure 6). These were done while the elbow was maintained in 90° of flexion. Sutures were anchored into remaining fascial structures and not over tightened, to avoid tissue necrosis and failure of the repair. The skin was closed, and the patient was placed in a padded long arm splint with the elbow flexed at 90° and neutrally rotated.

Four days postoperative, the patient was placed in a hinged elbow brace locked at 90°, and he was advised to refrain from weight loading. There was immediate resolution of his deformity and pain after surgery. At the initial rehabilitation visit, it was recommended to do self-assisted full flexion to 90° with full pronation and supination. Every 2 weeks thereafter, he progressed to 30° of extension, until full range of motion was achieved in 6 weeks.

The brace was removed 8 weeks after surgery, and assessment of the right elbow at that time was consistent with preoperative evaluation, 0° of extension and 140° of flexion with full pronation and supination.

Most recent evaluation of the patient occurred approximately 11 months postoperative. The range of motion at the right elbow was improved to +15° of extension to 150° of flexion. Monofilament testing disclosed intact median, radial, and ulnar sensation.

## Discussion

Isolated and intrasubstance ruptures of the short belly of the bicep are seldom reported.^4,5,8^ We describe a male patient who sustained an isolated intrasubstance tear of the short belly of the biceps brachii after a snow blower accident, with unique compression of the median and musculocutaneous nerves requiring microsurgical decompression for symptom relief. The injury was surgically corrected to a restoration of both range of motion and functional level, improvement in localized pain, resolution of positive Tinel sign, correction of deformity, and increased elbow strength.

Outside of military parachutists, biceps muscle belly tears of the short head are rare. Gilcreest analyzed 100 cases of biceps injury and reported two isolated ruptures of short head muscles: one partial and one complete.^4^ The mechanisms of injury were not described. Additional reports^6,9^ identified such an injury in water skiers resulting from sudden extension against a flexed elbow. Another study reported an isolated tear of the short belly of the biceps muscle in a patient whose arm was struck by a passing car door as it was abducted and externally rotated out of the window.^5^ Mizuno et al^10^ described an isolated short belly tear in a 20-year-old gymnast secondary to stress applied during an assumption of the gymnast crucifix position. There appears to be an association with these injuries and a rapid extending force against an eccentrically contracting muscle or a blunt blow to the muscle during contraction.^10^ As isolated short belly tears of the biceps brachii are rare, our case presents an example of this unique injury with compression of the median and musculocutaneous nerves.

The clinical features of biceps muscle belly rupture typically mimic those of the more common distal tendon rupture. On examination, patients will present with pain, tenderness, a palpable mass, ecchymosis, and weakness. Signs of blunt trauma and friction burns are common. A common finding specific to isolated traumatic rupture of the short head of the biceps is medial swelling and a medially oriented palpable defect. This defect may be difficult to see in the acute setting of partial tears^7^ and becomes more evident as initial swelling recedes.

Treatment of complete injury is usually surgical to restore adequate strength.^5^ Surgical decompression was warranted in our patient because both his musculocutaneous and median nerves were compressed by the injury. This case is unusual because median and musculocutaneous nerve decompression and internal neurolysis was required to relieve compression of each nerve. In the literature, only musculocutaneous nerve impingement has been reported for such injuries.^7^

## Conclusion

We present a patient with a rare intrasubstance tear of the short head of the biceps brachii uniquely entrapping both the median and musculocutaneous nerves. This case further establishes this rare injury pattern in the literature. Thorough physical examination and a high index of suspicion are keys to effective diagnosis and management.
